# **Self-aggregating** ***Lactiplantibacillus plantarum*** **enhances type-I interferon responses via the cytosolic sensors NOD2 and cGAS**

**DOI:** 10.1080/19490976.2026.2615490

**Published:** 2026-01-28

**Authors:** Selvin Solis, Elaina M. Maldonado, Subhankar Mukhopadhyay, Gwénaël Jan, José María Landete, Carlos Maluquer de Motes, Jorge Gutierrez-Merino

**Affiliations:** a School of Biosciences, University of Surrey, Guildford, UK; b School of Veterinary Medicine, University of Surrey, Guildford, UK; c School of Immunology & Microbial Sciences, King’s College London, London, UK; d STLO, INRAE, Institut Agro, Rennes, France; e Instituto Nacional de Investigación y Tecnología Agraria y Alimentaria (INIA-CSIC), Madrid, Spain

**Keywords:** *Lactobacillus*, probiotic bacteria, interferon, intracellular sensing, cGAS, NOD2

## Abstract

The gut microbiome plays a critical role in health, disease and immunity. To date, we have access to large datasets describing how the microbial diversity present in the gut correlates with many clinical conditions. However, the microbiome composition is taxonomically complex; influenced by many environmental factors; and variable between individuals and communities, thereby limiting functional and mechanistic insights into the microbiota‒host interactions. We are still unsure of the molecular mechanisms by which gut commensal microbes intrinsically possess to interact with the immune system and induce beneficial responses. This study has addressed this important question by revealing that only certain members of *Lactobacillaceae*, a bacterial family very well known for its probiotic properties, interact very intimately with macrophages because of their ability to simultaneously overexpress adhesive cell wall proteins and to self-aggregate, leading to significant production of type I interferon (IFN-I) cytokines. IFN-I cytokines are essential to confer protection against viral infections and auto-immune disorders. Specifically, we have proved that this enhanced IFN-I feature is strain-dependent and predominantly driven by cGAS, a molecule that activates the cytosolic sensor STING upon the recognition of bacterial DNA. Furthermore, another cytosolic sensor, NOD2, seems to be an additional stimulus to amplify IFN-I production, suggesting the involvement of successive molecular events for a prominent probiotic response. Our findings provide insight into how specific molecules of probiotic bacteria modulate or stimulate host responses, providing a better understanding of the molecular crosstalk between the microbiome and immune cells.

## Introduction

The interplay between the gut microbiome and the mucosal immune system affects human health in multiple ways, including the host natural defenses against infectious diseases and auto-immune disorders.^1^ Commensal bacteria are in constant communication with innate immune cells, which recognize microbial-associated molecular patterns (MAMPs) through pattern recognition receptors (PRRs), thus providing signals to maintain homeostasis and preventing entry and replication of pathogens.^2^ Although many commensals populate the host, only certain bacterial families, such as *Lactobacillaceae*, have been widely recognized as drivers of health-promoting functions in the gut and the respiratory and urogenital tracts.^3^ Extensive evidence shows that members of this family induce host protection;^4^ however, the molecular features that these bacteria intrinsically possess to stimulate and modulate the immune system remain largely unknown. It is very well known that PRRs and receptors that sense microbial molecules play pivotal roles in immunomodulation,^1^^,^^5^ but how microbes use their molecules to engage immune cells is a research question that has been overlooked. The lack of microbe-centric mechanistic approaches is a gap of knowledge that limits our ability to inform novel microbiome treatments and engineer a new generation of probiotics to maximize beneficial host responses.

Recently, our research group discovered that, unlike many other *Lactobacillaceae*, the species *Lactiplantibacillus plantarum* (LP) is detected by key innate immune cells such as macrophages via the Stimulator of Interferon Genes (STING),^6^ a cytosolic PRR that is activated upstream by the DNA-sensing receptor cyclic GMP–AMP synthase (cGAS)^7^ to initiate the production of type I interferons (IFN-Is) like interferon beta (IFN-β through the interferon regulatory factor 3 (IRF3) for the subsequent transcription of interferon stimulates genes (ISGs).^8^ ISGs are major protective effectors in innate immune responses at the mucosa, especially to combat viruses.^9^ LP has also been documented to confer protection against viral infections.^10^^,^^11^ These discoveries thus suggest the crucial role of intracellular sensing of probiotic bacteria for the activation of ISGs and the existence of specific molecular signatures that might contribute to homeostasis via IFN-I production.^6^ In this study, we have unveiled the IFN-I associated molecular signatures of LP, confirming that the immunomodulatory properties of *Lactobacillaceae* are not only genus but also strain dependent. Here, we report that the magnitude of the IFN-I responses activated by LP is regulated by its ability to bind to itself and interact with macrophages. The cell wall proteins of LP drive self-aggregation and potent macrophage interactions, resulting in the internalization of large quantities of bacterial cells and increased expression of ISGs that are intracellularly mediated by two cytosolic PRRs, cGAS and Nucleotide-binding oligomerization domain-containing protein 2 (NOD2).

## Results

### *Lactobacillaceae* that activate IFN-I responses show upregulated cell wall metabolism

**Figure 1.**
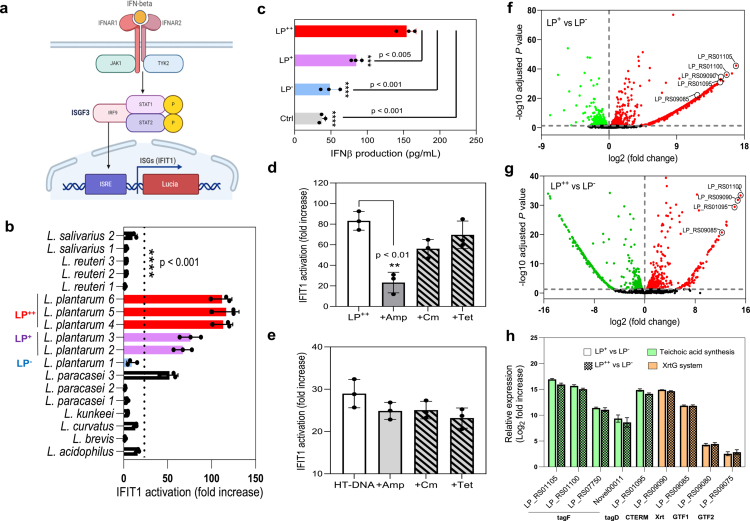
IFN-I responses are activated by strains of *L. plantarum* (LP) with a high cell wall metabolism profile. (a) PMA-differentiated THP1-IFIT1-Gluc macrophages express IFNAR1/2 receptors that sense IFN-*β* for the transcription of Gluc (Lucia) under the control of the IFIT1 promoter via the JAK1-STAT1 pathway. IFIT1 is an ISG whose promoter contains an interferon-stimulated response element (ISRE). (b) IFIT1 activation in THP-1 macrophages exposed to different species and strains of *Lactobacillaceae* at a ratio of 50 bacteria per macrophage over non-stimulated conditions (one-way ANOVA and Tukey’s multiple comparison; ****p < .001). Based on the observed IFIT1 activation levels, strains of LP were grouped into low (LP^−^, blue bar), moderate (LP^+^, purple bars), and high (LP^++^, red bars) inducers. (c) Production of IFN-*β* in macrophages after an 8 h challenge with LP^−^, LP^+^ or LP^++^. The results from the 3 challenges were compared with each other and an unstimulated control (Ctrl) condition (one-way ANOVA and Tukey’s multiple comparison i; **p < .01; ***p < .005). (d, e) IFIT1 activation in THP-1-IFIT1-GLuc macrophages exposed to LP^++^ previously treated with ampicillin (Amp), chloramphenicol (Cm) and tetracycline (Tet) at MICs of 0.5, 4 and 64 mg/mL, respectively (d), or transfected with HT-DNA in combination with the selected antibiotics at the given MIC concentrations (e). Data from each antibiotic treatment were statistically compared with their corresponding controls (LP or HT-DNA) using the Student’s t-test (**p < .01). (f, g) Volcano plots illustrating the genes differentially expressed in the LP^+^ (f) and LP^++^ (g) strains following a comparative RNA sequencing analysis against LP^−^. The upregulated and downregulated genes are indicated with red and green dots, respectively, and within the most upregulated genes, transcripts associated with cell wall metabolism were identified in both LP^+^ and LP^++^. (h) Relative expression of cell wall-associated transcripts in LP^+^ and LP^++^ compared with LP^−^. The expression of the selected genes is presented as a log2-fold increase, and these genes encode enzymes involved in the synthesis of teichoic acids (green) and sortase-dependent proteins (orange).

To expand on the results of our previous publication,^6^ a total of 21 strains of *Lactobacillaceae*, including 8 different genera and species (Extended Data Table 1), were screened based on their ability to trigger the activation of the interferon-induced protein with tetratricopeptide repeats 1 (IFIT1) in human THP-1 macrophages (Figure 1a). IFIT1 is an ISG whose expression can also be directly activated by IRF3.^12^ THP-1 monocytes expressing GLuc under the control of the IFIT1 promoter^13^ were differentiated for 48 h and subsequently exposed to the selected *Lactobacillaceae* strains. GLuc activity was measured in the media and is presented as the fold increase over non-stimulated macrophages. Amongst all the genera and species tested, only *Lactiplantibacillus plantarum* (LP) and *Lacticaseibacillus paracasei* induced significant IFIT1 activation that ranged from moderate to high (Figure 1b), especially in response to LP (purple and red bars in Figure 1b). Based on this gradual IFIT1 response, which clearly different amongst the LP strains, these strains were categorized into 3 groups: low (LP^−^), moderate (LP^+^) and high (LP^++^) IFN-I inducers; and from each group, the following strains were selected as their representatives: P5^14^ (LP^−^), WCFS-1^15^ (LP^+^) and EML1^16^ (LP^++^). The differential IFN-I production in response to these 3 LP strains was confirmed using an ELISA to detect human IFN-β (Figure 1c). LP^++^-challenged THP1 macrophages secreted significantly more IFN-β than those exposed to LP^+^ (*p* < 0.005) or LP^−^ (*p* < 0.001) at 8 h post-challenge. To determine whether IFN-I responses require metabolically active LP cells, we treated the selected LP strains with minimum inhibitory concentration (MIC) of the following antibiotics: ampicillin, chloramphenicol, and tetracycline (Figure 1d). Whilst chloramphenicol and tetracycline inhibit protein synthesis, ampicillin prevents cell wall synthesis. Treatment with ampicillin significantly reduced the capacity of the LP^++^ (*p* < 0.01), but not the HT-DNA, to activate IFIT1 (Figure 1d,e), suggesting that an intact cell wall is important for bacteria-induced IFN-I responses. We then conducted comparative transcriptomics using RNA from the mid-exponential phase of the 3 LP cultures. Compared with LP^−^, which does not induce IFN-I responses, the GO enrichment analysis revealed an over-representation of membrane/cell wall biological processes in both LP^++^ and LP^+^ (Extended Data Figure 2). In particular, we observed notable over-expression of transcripts encoding markers associated with cell wall metabolism (Figure 1f,g and Extended Data Excel file 1), such as sortase-dependent proteins and enzymes involved in the synthesis of teichoic acids (Figure 1h), including four CDP-glycerol glycerophosphotransferases (tagF) [LP_RS01105, LP_RS01100, LP_RS07750, Novel00011], one glycerol-3-phosphate cytidylyltransferase (tagD) [LP_RS01095] and a complete Exosortase G (XrtG) system composed of an exosortase-dependent Firmicu-CTERM sorting domain-containing protein (CTERM) [LP_RS09090), an exosortase G family protein (Xrt) [LP_RS09085] and two XrtG-exosortase-associated glycosyltransferases (GTF1-2) [LP_RS09080, LP_RS09075]. Our initial screening therefore revealed that the induction of IFN-I responses by *Lactobacillaceae* is genus- and strain-dependent and that the difference in these responses correlates with cell wall integrity and metabolism.

**Figure 2.**
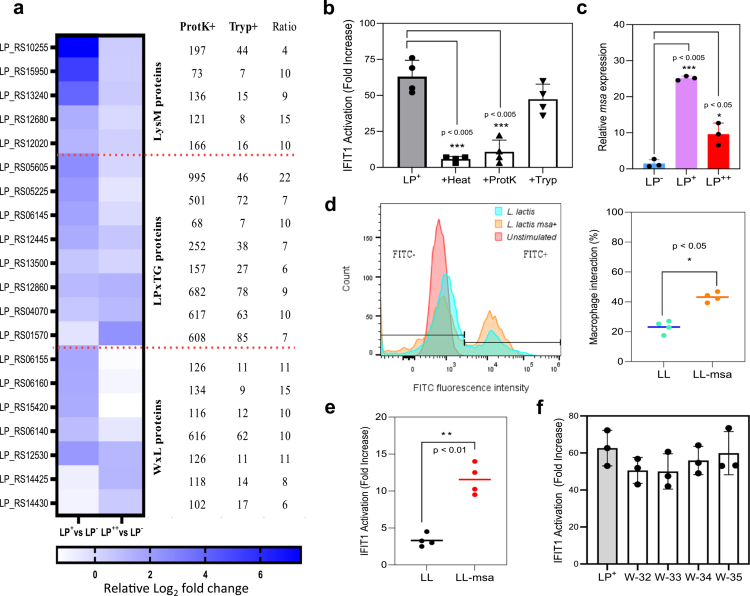
Adhesive properties of *L. plantarum* (LP) are important for initiating IFN-I responses. (a) Heatmap illustrating the relative expression of genes encoding cell wall adhesins with LysM, LPxTG and WxL domains in LP^+^ and LP^++^, when compared to LP^−^. The number of predicted proteinase K and trypsin cleavage sites and their corresponding ratios that are listed on the right were obtained using the Expasy PeptideCutter tool. (b) IFIT1 activation in IFIT1-GLuc THP-1 macrophages exposed to LP^+^ pretreated with proteinase K or trypsin at 100 and 25  µg/mL, respectively, for 1 h at 37 °C. The heat-treated LP strains were used as a loss of cell wall integrity control, and the data from each treatment were statistically compared with those from the untreated LP control using the Student’s t-test (***p < .005). (c) Relative expression of the mannose-specific adhesin (*msa*) gene (LP_RS05225) in LP^+^ and LP^++^ over comparison with that in LP^−^. Gene expression is presented as a relative fold change over non-stimulated conditions using *gapB* (GAPDH) as the housekeeping gene and Student’s t-test (*p < .05; ***p < .005). (d) Percentage of THP-1 macrophages that interact with FITC-labelled cells of *L. lactis* (LL) and msa-expressing LL (LL-msa) as illustrated with a histogram and quantified by flow cytometry Student’s t-test (*p < .05). (e) IFIT1 activation in THP-1-IFIT1-GLuc macrophages challenged with at a ratio of 50 cells LL or LL-msa cells per macrophage for 2 h and statistically compared as a fold increase over a non-stimulated condition using Student’s t-test (**p < .01). (f) IFIT1 activation in IFIT1-GLuc THP-1 macrophages exposed to highly msa-expressing LP^+^ variants (W-32-35) at a ratio of 1:50 and presented as a fold increase over a non-stimulated condition. No significant differences were recorded when compared to macrophages challenged with LP+  (Student’s t-test).

### Cell wall adhesins of LP are key factors to elicit IFN-I responses

To shed some light on the cell wall differences observed between the three LP strains, we first investigated the role that adhesins play in bacterium‒macrophage interactions. Adhesins with the LPxTG, LysM or WxL domains anchored to the cell wall peptidoglycan have been reported to possess binding abilities.^17^^,^^18^ As illustrated in Figure 2a, the LP^+^ and LP^++^ strains express a significant number of LPxTG/LysM/WxL adhesins at a much higher level than LP^−^. All these adhesins show multiple proteinase K cleavage sites and a proteinase K/trypsin ratio in favor of proteinase K. In fact, we found that proteinase K treatment significantly decreases the capacity of LP^+^ to activate IFIT1 (*p* < 0.005), whereas treatment with trypsin failed to do so (Figure 2b). To verify the hypothetical involvement of adhesins in binding to macrophages, we focused our attention on LP_RS05225, a gene encoding an LPxTG adhesin referred to as mannose-specific adhesin (msa), which is known to promote adhesion to human cells.^19^ qPCR analysis confirmed that *msa* is significantly upregulated in the LP^+^ and LP^++^, when compared to LP^−^ strain (*p* < 0.005 and 0.05, respectively) (Figure 2c). We then conducted a gain of function test using a recombinant strain of *Lactococcus lactis*^20^ (LL) transformed with a plasmid expressing msa (LL-msa)^19^ (Extended Data Table 1). LL lacks prominent adhesins and is a reliable host for the heterologous expression of recombinant proteins.^21^ Using FITC labeling and a flow cytometry (FC) gating strategy (Extended Data Figure 3), more THP1 macrophages were observed to interact with LL-msa than with LL (*p* < 0.05) (Figure 2d). More importantly, LL-msa induced a significant increase in IFIT1 activation compared with the non-recombinant LL strain (*p* < 0.01) (Figure 2e). Therefore, the expression of adhesins such as msa elicits IFN-I signaling and enhances the interaction between LP and macrophages. However, the use of variants of LP^+^ that generate more *msa* transcripts than LP^+^ (W-32-35, Extended Data Table 1)^19^ was unsuccessful in triggering significantly higher IFN-I activation (Figure 2f), suggesting that additional IFN-I elicitors from the LP. Thus, adhesins may be important to initiate and enhance interaction with macrophages, thereby influencing the IFN-I responses; however, adhesins might remain insufficient as a sole factor to increase the magnitude of these responses.

**Figure 3.**
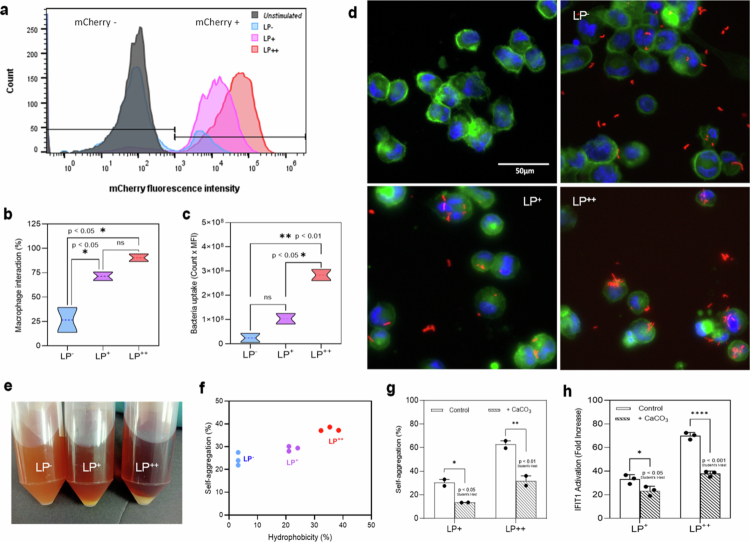
Self-aggregation of *L. plantarum* (LP) enhances internalization by macrophages. (a–c) Flow cytometry analysis of the interaction between THP-1 macrophages and LP strains expressing mCherry at 2 h post-LP exposure as illustrated with a histogram overlay plot (a) and quantified based on the levels of macrophage interaction (b) and bacteria uptake (c). Comparative statistical analysis was conducted using one-way ANOVA and Tukey’s multiple comparison (*p < .05; **p < .01). (d) Immunofluorescence microscopy showing the internalization of mCherry-expressing LP^−^, LP^+^ and LP^++^ strains (red) in THP-1 macrophages stained for their nucleus (Hoechst, blue) and F-actin (CellMask™ Actin Tracking dye, green). Images were acquired using the blue–green-far red channels and merged at a 20x objective. (e, f) Overnight cultures of LP^−^, LP^+^ and LP^++^ showing differences in their self-aggregation ability (e) and the correlation between the percentages (%) of self-aggregation and cell surface hydrophobicity for each LP strain as determined by their sedimentation ability and their adhesion to the organic solvent xylene, respectively. (g, h) The self-aggregation differences observed in the LP strains when pre-cultured in media with or without CaCO_3_ supplementation (g) correlate positively with their ability to activate IFIT1 in THP-1 macrophages (h). Differences in self-aggregation and IFIT1 activation were statistically compared using Student’s t-test (*p < .05; **p < .01; ****p < .001).

### Self-aggregation of LP results in an increased macrophage uptake

Building upon the binding role of adhesins, we proceeded to quantify the levels of interaction of THP-1 macrophages with the LP strains previously transformed with an mCherry-expressing plasmid.^22^ We calculated the number of macrophages interacting with LP (macrophage interaction) and vice versa (bacteria uptake) by FC based on the frequency and count of mCherry-positive single cells and their median fluorescence intensity (MFI) (Figure 3a–c). Similarly, the number of macrophages interacting with LP^++^ was higher than that interacting with the LP^+^ and LP^−^ strains (*p* < 0.05), with % values ranging between 80–100, 60–80 and 20–50, respectively (Figure 3b). Additionally, we observed that macrophages exposed to LP^++^ contained many more bacteria than those challenged with LP^−^ and LP^+^ (*p* < 0.01 and 0.05) (Figure 3c), which was confirmed by immunofluorescence microscopy (IM) (Figure 3d). LP^++^ was internalized as much larger aggregates when compared to LP^+^ and LP^−^. Furthermore, the self-aggregation ability of the LP strains was clearly visualized in overnight cultures, with an evident aggregation pattern towards those derived from LP^+^ and LP^++^ (Figure 3e). In particular, the percentage of self-aggregation in LP^++^ was significantly higher than that of LP^−^ as determined by their ability to sediment; and this prominent self-aggregation capacity was positively correlated with their cell surface hydrophobicity, as quantified by the levels of adhesion to the organic solvent xylene (Figure 3f). The self-aggregation and hydrophobicity of LP^+^ cells are also higher than those from LP^−^ cells, but are less significantly different if compared to LP^++^. To determine the role of bacterial aggregation in macrophage interactions and IFN-I signaling, the LP strains were cultured under pH-controlled conditions using CaCO_3_ as an acid neutralizer. The aggregation of *Lactobacillaceae* is associated with a decrease in pH by sugar fermentation.^23^ LP^+^ and LP^++^ showed a significant reduction in their self-aggregation levels in media supplemented with CaCO₃ at 0.2% (*p* < 0.05 and 0.01) (Figure 3g) that coupled with reduced IFIT1 activation in LP-challenged macrophages (Figure 3h). Thus, our findings indicate that bacterial self-aggregation promotes engulfment by macrophages at any given point of contact, and this internalization of the LP mass results in increased levels of IFN-I responses. These results were also confirmed with foetal-mice liver derived Max Plank Institute (MPI) macrophages (Extended Data Figure 4), which engage with the LP strains exactly as the THP-1 macrophages did.

**Figure 4.**
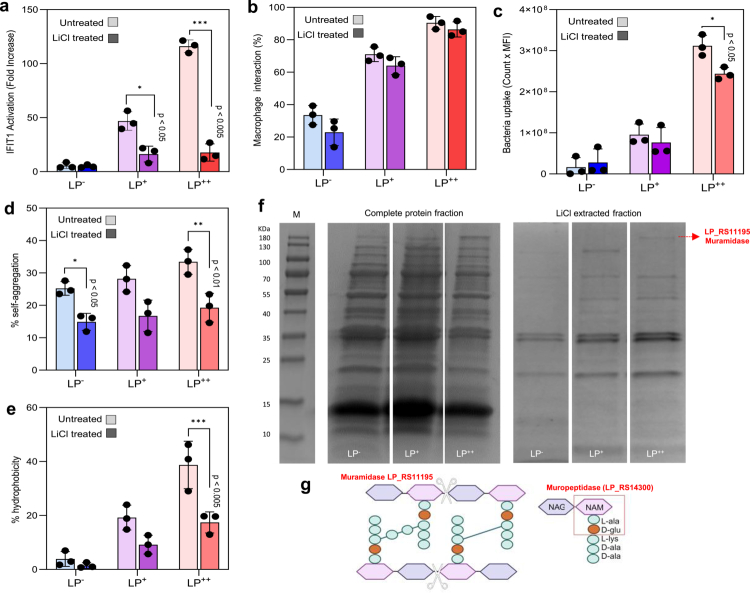
Cell wall proteins of *L. plantarum* (LP) are essential for self-aggregation and enhanced IFN-I responses. (a) IFIT1 activation in IFIT1-GLuc THP-1 macrophages exposed to LP^−^, LP^+^ or LP^++^ previously treated with lithium chloride (LiCl) at 5 M. (b, c) Quantification of the macrophage interaction (b) and bacterial uptake (c) by flow cytometry following a 2 h challenge with untreated or LiCl-treated mCherry-expressing strains of LP^−^, LP^+^ and LP^++^. (d, e) Levels of self-aggregation (d) and cell surface hydrophobicity (e) for each LP strain previously treated with LiCl compared with their corresponding controls (untreated LP). Data from each LiCl treatment were statistically compared with their corresponding controls (untreated LP) using the Student’s t-test (*p < .05; **p < .01; ***p < .005). (f) Readyblue™ stained SDS–PAGE gels showing the selectivity of the LiCl protein extraction from LP^−^, LP^+^ or LP^++^ cells when compared to that with the B-PER™ reagent for complete protein extraction. The muraminidase LP_RS11195 was identified only in the LiCl-treated extracts from LP^++^ by in-gel trypsin digestion and nano-LC-MS/MS. (g) Illustration of how the enzymatic activity of muramidases such as LP_RS11195 on the cell wall peptidoglycan structure (scissors) results in the formation of muropeptides consisting of N-acetylglucosamine (NAG)-N-acetylmuramic acid (NAM) disaccharides attached to a peptide chain of 5 amino acid residues. Further enzymatic activity by muropeptidases such as LP_RS14300 generates the NOD2 ligand muramyl dipeptide (MDP) (red square).

### Cell wall-derived proteins of LP associate with self-aggregation and induce IFN-I responses in macrophages

To confirm the key involvement of the cell wall proteins of the LP in the level of the IFN-I responses observed, we treated the LP with lithium chloride (LiCl). LiCl has been employed in many studies to specifically extract and solubilize non-covalently bound proteins from *Lactobacillaceae* without compromising the cell wall structure.^24^ Some of these proteins include cell wall proteins with LysM and WxL domains that are associated with adhesion and aggregation properties.^25^ We first compared the levels of IFIT1 activation between macrophages stimulated with 5 M LiCl-treated- and untreated-LP strains (Figure 4a) and found that the LiCl treatment significantly decreases the capacity of LP^+^ and LP^++^ to induce IFIT1 activation (*p* < 0.05 and 0.005). On the other hand, when interactions between LP and macrophages, and vice versa (bacteria uptake), were evaluated by FC (Figure 4b,c), LiCl treatment slightly reduced the LP‒macrophage interaction (Figure 4b) but was not significantly different if compared with the control using untreated LP cells. However, bacterial uptake decreased significantly after the LiCl treatment, especially in macrophages exposed to LP^++^ (*p* < 0.05) (Figure 4c). These results indicate that the proteins extracted might not be crucial for macrophage interaction, but could nevertheless be essential to enhance internalization of LP by macrophages. In fact, this reduction in the LP uptake upon LiCl treatment correlated positively with a reduction in the self-aggregation and cell surface hydrophobicity of LiCl-treated LP cells (*p* < 0.05, 0.01 and 0.005) (Figure 4d,e). To confirm the contribution of the LiCl-extracted proteins to the self-aggregation of LP and the subsequent increase in bacterial uptake, these proteins were visualized on an SDS‒PAGE gel (Figure 4f). Protein bands from LP^++^, which were more intense on the gel, were then excised and subjected to in-gel trypsin digestion and the resulting peptides were analysed by nanoscale liquid chromatography coupled with tandem mass spectrometry (nano LC‒MS‒MS/MS) (Extended Data Excel file 2). The whole LiCl extracts were also subjected to nano-LC‒MS/MS analysis. Overall, the intensity of the protein bands was higher in the LiCl extracts derived from LP^+^ and LP^++^, and among the identified proteins, those of interest were a muramidase (LP_RS11195 in Figure 4a,f) protein with a LysM domain (LP_RS14300), listed as a muropeptidase, which were only found in the LiCl extracts of LP^++^. Interestingly, both muramidases and muropeptidases contribute to the generation of muramyl dipeptides (MDPs) that are known to act as ligands for intracellular receptors such as NOD2^26^ (Figure 4g). Thus, our results suggest that the combination of aggregative proteins such as those with Lys-M domains and MDP-generating enzymes such as muramidases and muropeptidases are vital to initiate an optimal intracellular sensing of LP that leads to prominent IFN-I activation. We then investigated the cytosolic sensors that are essential to drive enhanced IFN-I responses in LP-challenged macrophages.

### Self-aggregating LP enhances canonical IFN-I responses via the cGAS-STING axis and NOD2

**Figure 5.**
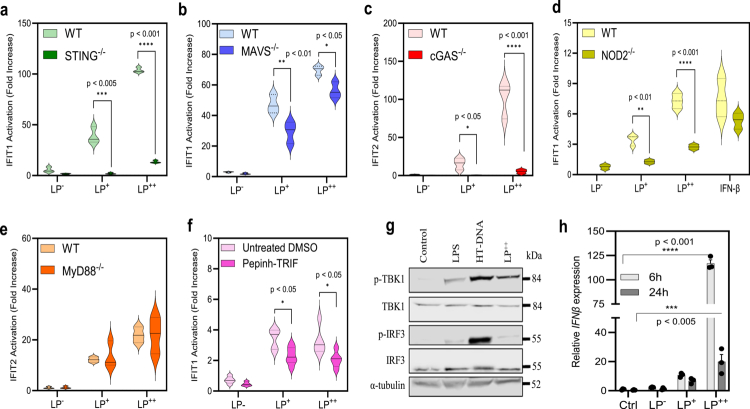
Internalization of *L. plantarum* (LP) in macrophages induces an IFN-I signaling process that is mediated by the cytosolic sensors cGAS and NOD2, leading to an early activation of IRF3 and IFN-β production. (a–e) IFIT1/2 activation in THP1-IFIT1-Gluc (or IFIT2-Lluc) macrophages and their derivative cell lines deficient ^(^^−/−)^ for STING (a), MAVS (b), cGAS (c), NOD2 (d) and Myd88 (e) previously exposed to LP^−^, LP^+^ and LP^++^ at a 1:50 ratio. Data between the original cells (WT) and their corresponding knockout (STING/MAVS/cGAS) or knockdown (NOD2) cells were statistically compared using the Student’s t-test (**p < .01; ****p < .0001). IFN-*β* was used as a positive control. (f) IFIT-1 activation in LP-challenged THP1-IFIT1-Gluc macrophages treated with the TRIF inhibitor Pepinh-TRIF in DMSO or DMSO (mock) (Student’s t-test; *p < .05). (g) Immunoblot analysis of phosphorylated TBK1 (*p*-TBK1), phosphorylated IRF3 (*p*-IRF3), total TBK-1 and total IRF3 in THP-1 macrophages exposed to either LP^++^ or LPS or transfected with HT-DNA for 2 h. (h) Relative expression of the *IFN-β gene* in THP-1 macrophages at 6 and 24 h post-challenge with LP^−^, LP^+^ or LP^++^. Gene expression is presented as a relative fold change over non-stimulated conditions using *gapB* (GAPDH) as the housekeeping gene (Student’s t-test; *p < .05; ***p < .005).

Our previous studies revealed that the cytosolic sensors STING and, to a much smaller degree, MAVS play a prominent role in the activation of IFN-I responses after LP internalization.^6^ This observation has been confirmed in this study (Figure 5a,b), yet with a clear dependency on the LP strain used. However, this poses the question of whether IFN-I production derives from the recognition of DNA or any other MAMPs of the LP. STING activation is mediated not only by mammalian 2’3’-cGAMP, the product of activated cGAS,^27^ but also by bacterial cyclic dinucleotides (CDNs).^28^ We therefore used THP1 cells lacking cGAS. As illustrated in Figure 5c, the IFIT1 activation triggered by LP^+^ and LP^++^ was abolished in the absence of cGAS (*p* < 0.05 and 0.001), ruling out the contribution of bacterial CDNs. Additionally, we tested whether other cytosolic sensors are involved in the recognition of LP and the activation of IFN-I responses. As described in the previous section, the removal of MDP-generating enzymes from the cell wall of LP^++^ resulted in very low IFN-I responses; thus, we proceeded to generate THP1-IFIT1 macrophages in which NOD2 was knocked down via siRNA transfection (Extended Data Figure 5). When compared to the LP-challenged control macrophages (transfected with a non-targeting negative control siRNA), the siNOD2 macrophages showed a significant decrease in their ability to activate IFIT1 after being exposed to the LP strains (*p* < 0.01 and 0.001) (Figure 5d). Simultaneously, we proved that the hypothetical involvement of endosomal TLRs as IFN-I elicitors^29^ is minimal or non-existent (Figure 5e,f). The absence of the myeloid differentiation primary response 88 (MyD88) in THP-1 cells does not alter the levels of IFIT1 activation upon exposure to LP (Figure 5e), and although the inhibition of the TIR domain-containing adapter-inducing interferon-*β* (TRIF) results in a significant decrease in the IFIT1 expression (*p* < 0.05) (Figure 5f), this represents only a small reduction (25%) by comparison with that observed in cells deficient for cGAS (almost 100%) or NOD2 (approximately 60%). TRIF is an essential adaptor protein for TLR3, a PRR that recognizes RNA.^29^ Taken together, these results indicate that the engulfment of self-aggregating LP cells by macrophages triggers potent IFN-I responses that are mainly mediated by the DNA sensor cGAS and, to some extent, NOD2.

**Figure 6.**
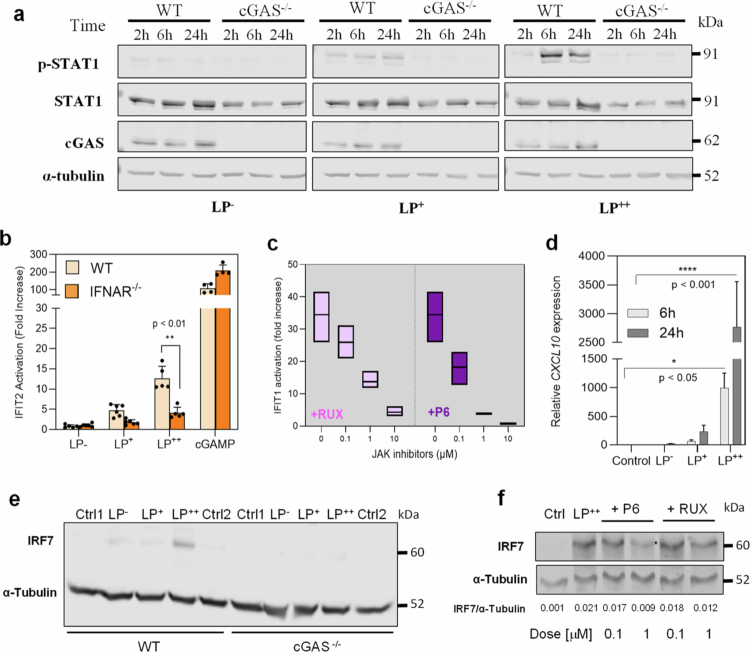
The IFN-I signaling process triggered by *L. plantarum* (LP) induces late activation of ISGs and IRF7 via the JAK-STAT1 pathway. (a) Immunoblot detecting phosphorylated STAT1 (*p*-STAT1) and total STAT1 in THP-1 macrophages and their derivative cGAS knockout (KO) macrophages after 2, 6 and 24 h post-exposure to LP^−^, LP^+^ and LP^++^. (b) IFIT2 activation in LP-exposed THP1-IFITs-Lluc macrophages and their derivative IFNAR knockouts ^(^^−/−)^ at a macrophage-LP ratio of 1:50. Data between the original cells (WT) and the IFNAR knockouts were statistically compared using the Student’s t-test (**p < .01). The potent cGAS-derived STING agonist cGAMP was used as a positive control for both cell lines. (c) IFIT1 activation in THP-1-IFIT1-GLuc THP-1 macrophages exposed to LP^++^ and the JAK1 inhibitors 6P and RUX at increasing concentrations (0–10 µM). (d) Relative expression of the CXCL10 gene in THP-1 macrophages at 6 and 24 h post-challenge with the LP strains. Gene expression is presented as a relative fold change over non-stimulated conditions using *gapB* (GAPDH) as the housekeeping gene (Student’s t-test; *p < .05; ****p < .001). (e, f) Immunoblots detecting IRF7 in THP-1-IFIT2-Lluc THP-1 macrophages and their corresponding cGAS KO cells after 12 h of challenge with the LP strains (e) or LP^++^in combination with 6P and RUX at 0.1 and 1 µM (f). *α*-Tubulin is the loading control for both immunoblots and the housekeeping protein for the calculation of the IRF7/α-tubulin ratios indicated for each of the JAK1 inhibitors’ concentrations.

To verify the association between self-aggregation and high IFN-I responses, we observed that only LP^++^ triggers an early phosphorylation of the canonical TANK-binding kinase (TBK1)-IRF3 pathway in the THP-1 macrophages (Figure 5g) that leads to very high levels of IFN-*β* expression as quantified at 6 h post-challenge (*p* < 0.001) (Figure 5h). With the other strains (LP^−^ and LP^+^), the phosphorylation of TBK1 and IRF3 was not visualized by immunoblotting, and the expression of IFN-*β* was significantly lower. Subsequently, we explored the activation of the signal transducer and activator of transcription 1 (STAT1), a primordial IFN-I signal mediator that is phosphorylated via the type-I IFN receptor (IFNAR) and Janus kinase 1 (JAK1).^30^ We observed that the levels of STAT1 activation was positively correlated with the ability of LP to self-aggregate and activate IFN-I responses, and as expected, this signal decreased in cells deficient for cGAS^29^ (Figure 6a). To reinforce the activation of the JAK1-STAT1 pathway, we determined IFIT1 activation in LP-challenged THP-1 macrophages lacking IFNAR or that were further treated with the JAK1 inhibitors pyridone 6 (P6) and Ruxolitinib (RUX) at increasing concentrations (Figure 6b,c). The absence of IFNAR resulted in a significant reduction in IFIT1 activation (*p* < 0.01) (Figure 6b), as the exposure to both inhibitors did in a dose-dependent manner (Figure 6c). Furthermore, we recorded late expression of the prototypical ISG CXCL10 in LP-stimulated macrophages, which significantly increased at 24 h post-challenge, particularly with LP^++^ (*p* < 0.001) (Figure 6d), confirming the downstream effect of the JAK1-STAT1 activation. Interestingly, we have also recorded a late over-production of the Interferon Regulatory Factor 7 (IRF7) in LP^++^ exposed macrophages (Figure 6e), which in the context of the JAK1-STAT1 pathway suggests a paracrine-autocrine positive feedback loop mediated by the canonical STAT1-STAT2-IRF9 signalling complex.^29^ cGAS deficiency and treatment with P6 and RUX were found to repress the expression of IRF7 (Figure 6e,f).

## Discussion

Innate immune cells produce IFN-I cytokines upon recognition of MAMPs, resulting in important, protective responses via the synthesis of ISGs.^8^^,^^30^ Although many studies have focused on how pathogenic microbes deceive or take advantage from IFN-I activation, the role that beneficial microbes play in that activation has also recently gained much attention.^31^ In particular, it has been shown that *Lactobacillaceae* are able to induce the production of IFN-I via endosomal TLRs^32^^,^^33^ and nucleic acid cytosolic sensors.^6^ Here we demonstrate that these beneficial IFN-I responses depend on the genus of *Lactobacillaceae* and that DNA sensing is the main intracellular mechanism mediating those responses. Among all the genera and species selected in this study, *Lactobacillus plantarum* (LP) has clearly been identified as a prominent IFN-I inducer through the cGAS‒STING axis. Other genera, such as *Lacticaseibacillus* and its species *L. paracasei* (this study) and *L. rhamnosus*^34^ could also be included in the same list, and interestingly, this cGAS-dependent IFN-I activation seems to be influenced not only by the genus or species but also by the strain. Only LP strains capable of exerting simultaneously binding properties and self-aggregation are associated with potent ISG activation.

LP possesses a very nomadic lifestyle and remarkable metabolic flexibility.^35^ Among the properties that make LP such an adaptable species capable of binding to eukaryotic cells^36^ and self-aggregating.^37^ In this study, we confirmed that these two cell wall-associated properties are strain dependent and that both are essential for initiating an IFN-I signaling cascade. Within the context of probiotics, this finding highlights a novel concept linking cell wall functionality with immunomodulation. Adhesion has always been shown to favor probiotic features,^38^ but self-aggregation has normally been associated with biofilm formation.^39^ In this respect, we have observed that the presence of an adhesin (msa), a Lys-M domain muropeptidase (LP_RS14300) and a muramidase (LP_RS11195) is related to high IFN-I responses. Strains that highly express msa interact more intimately with macrophages, but without the muropeptidase and muramidase, this bacteria‒macrophage interaction results in a moderate production of IFN-I cytokines. Our data indicate not only that without initial, strong binding between LP and macrophages, low IFN-I responses are bound to occur but also that increased msa expression does not necessarily result in increased IFN-I responses. LP strains harboring specific muropeptidases and muramidases tend to form more aggregates, activating much higher IFN-I responses, and this correlation is attributed to the massive internalization of bacterial cells by macrophages.

In our previous work, we confirmed that LP cells must be phagocytosed by macrophages in order to induce an IFN-I response.^6^ However, the precise mechanisms by which bacteria are internalized and processed once they are inside macrophages remain to be elucidated. Here, we shed some light on this question by revealing that two cytosolic sensors, NOD2 and cGAS, engage with MAMPs. On the one hand, we have shown that cell wall muramidases and muropeptidases are likely important for initiating the intracellular sensing of LP and subsequent IFN-I production. These enzymes hydrolyse the peptidoglycan layer of *Lactobacillaceae*, releasing NOD2 ligands such as MDP.^40^ In our macrophage model, we have observed that NOD2 deficiency significantly decreases the ability of LP to activate IFN-I responses, and this action was mirrored in the absence of LP_RS14300 and LP_RS11195 within the LP cell wall. On the other hand, cGAS is essential for an effective IFN-I production via the TBK1-IRF3 pathway, as previously reported.^41^ How cGAS and NOD2 interact to activate prominent IFN-I responses is unclear.^42^ However, one point of nexus is the fact that upon bacterial internalization, NOD2 cooperates with IRF3 for the co-production of IFN-I cytokines (e.g. IFN-α and IFN-β) through the activation of the TBK1/IRF5 pathway.^43^ Furthermore, although our results clearly show that cGAS is crucial for triggering high IFN-I responses, we should not ignore the involvement of other DNA sensors that activate the production of IFN-I cytokines through the TBK1-IRF3 pathway, such as DDX41, IFI16 and ZBP1.^44^ These observations introduce additional factors to consider for future investigations and possible explanations on why the magnitude of the IFN-I responses observed in this study is strain dependent. The bacterial cargo engulfed by macrophages is likely degraded within the phagolysosome, leading to the release of MAMPs such as MDP and DNA^45^ that are subsequently sensed by NOD2, cGAS and/or other DNA sensors. The more cargo that is engulfed, the more activation of IRF5 and IRF3, which is therefore expected to be triggered. We know that NOD2 directly interacts with phagolysosomes,^26^ but whether the size of the cargo may lead to their destabilization for DNA release in the cytosol is a very recent scientific question^46^ that deserves further studies. How efficiently macrophages are at internalizing and processing LP would also be worth exploring, as this might be affected by specific strain features.

Our data indicate that the cytosolic presence of LP material, presumably DNA, is sensed by cGAS, triggering the production of IFN-β and the subsequent activation of the ISG CXCL10 through the JAK1-STAT1 pathway. Previously, we reported the production of additional ISGs in the form of MxA and OAS1 in LP-exposed macrophages. Thus, CXCL10 activation observed in this study, amongst other beneficial attributes,^47^ reinforces the potential anti-viral properties of LP.^10^ Furthermore, we have observed that the cGAS-JAK1-STAT1 cascade results in late overexpression of IRF7, suggesting the involvement of a paracrine-autocrine IFN-I feedback loop via the trimerized complex STAT1–STAT2–IRF9, also known as Interferon Stimulated Gene Factor 3 (ISGF3).^48^ Nevertheless, we cannot rule out the contribution of other STAT dimers and/or STAT-independent pathways as well as additional IFN cytokines (type III IFN-λ) to the observed ISG activation. CXCL10 responds to IFN-α/β and IFN-λ ^49^^,^^50^ and can be activated by tyrosine-protein kinase 2 (TYK2)-dependent pathways, such as mitogen-activated protein kinases (MAPKs) and the phosphoinositide 3-kinase (PI3K) pathway.^51^ However, we have shown that 6P, an inhibitor of JAK1 and TYK2, completely blocks the observed IFN-I responses in LP-exposed macrophages. Furthermore, IFN-λ is produced following the activation of IRF3 and IRF7,^50^ and we found that both IRFs are activated following the internalization of LP. Further experiments with cell lines and/or mouse models that are deficient for different ISG-related markers might be a reasonable starting point to fully complete the map of all the up- and down-stream pathways that the intracellular sensing of LP activates in macrophages.

### Strengths and limitations

Our results highlight the relevance of studying host‒microbe interactions not only from the bacterial perspective but also as a cascade of interlinked events. We have proved that the number of probiotic bacterial cells entering macrophages depends on their ability to self-aggregate, but without adhesive proteins (e.g msa) that help probiotics bind to the macrophages, their internalization becomes irrelevant. On the other hand, the presence of certain cell wall proteins (e.g. muramidase and muropeptidase) could be crucial for enhancing self-aggregation as well as facilitating the activation of cytosolic sensors such as NOD2. Although the intracellular sensing of probiotic bacteria such as LP seems to be primarily mediated by cGAS, without the contribution of NOD2, the magnitude of the IFN-I responses would remain moderately high instead of extremely high. The generation of mutants of LP that are deficient in msa, muramidases and muropeptidases could help clarify their functionality, but as a considerable number of cell wall proteins have been associated with adhesion and aggregation in LP,^36^^,^^37^ multiple deletions might be needed to verify specific phenotypes. The elimination of one protein could even be related to several functions, as we have observed with the muramidase and muropeptidase. Furthermore, it is important to note that the enhanced IFN-I responses that we report here were observed using THP-1 reporter cell lines and murine-derived MPI macrophages. Although the homogeneous genetic background of these cell lines reduces phenotypic variability, thereby enhancing reproducibility compared to the donor-dependent variation observed in primary cells, the validation of our mechanistic findings might require the use of primary cells and/or in vivo models to draw more definitive conclusions. Nevertheless, our previous work validated our in vitro THP-1 cell model by demonstrating that LP induces IFN-I responses in human PBMCs.^6^

## Conclusions

**Figure 7.**
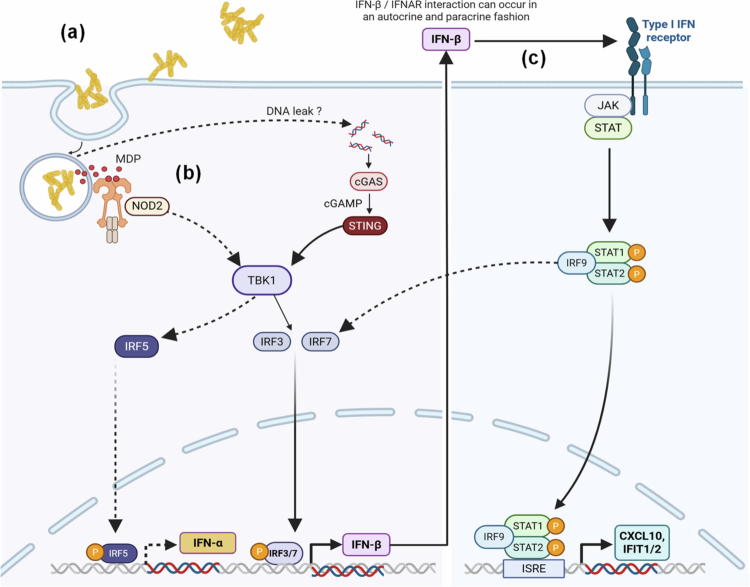
Schematic representation of the IFN-I signaling events that self-aggregating strains of *L. plantarum* (LP) activate in macrophages. (a) Adhesion of LP to macrophages and the subsequent internalisation of aggregates into phagosomes. (b) Release of MAMPs such as MDP and DNA into the cytosol activates the intracellular PRRs NOD2 and cGAS to consequently trigger the activation of IRF5 and IRF3, respectively. (c) Production of IFN-*β* is sensed by the IFNAR receptors, resulting in the overexpression of ISGs through the JAK1-STAT1 pathway and an IRF7-mediated paracrine-autocrine IFN-I feedback loop. The solid lines indicate signaling events identified in this study, and the broken lines indicate pathways hypothesized or previously indicated in the literature. Figure created with Biorender.

Overall, our findings provide a novel perspective on the interactions between probiotic bacteria and human cells, with further implications in the use and manipulation of probiotics to modulate innate immune responses. This study has identified key phenotypical features of the prototypical probiotic bacterium LP that are essential for the activation of enhanced IFN-I responses in macrophages. These features include adhesion and self-aggregation, and although self-aggregation seems to be the primordial factor regulating high IFN-I responses, both cooperate to stimulate phagocytosis. Following phagocytosis, the bacterial cargo is then recognized by the cytosolic sensors NOD2 and cGAS, resulting in a substantial production of IFN-I cytokines and the inevitable activation of ISGs. The succession of these interdependent events are vital for successful IFN-I responses (Figure 7), which makes the identification of IFN-I-associated molecules very difficult. However, we have been able to identify several potential immunomodulatory molecules, including an adhesin for binding (msa) and two NOD2 ligand-generating enzymes (a muropeptidase and a muramidase) that could be associated with self-aggregation. These molecules are not exclusive to LPs; thus, their presence in other commensal bacteria might inform IFN-I-focused gut microbiome therapies and help the scientific community in the search for new beneficial bacteria. High IFN-I production could be beneficial for combatting viral infections and bowel cancer, but dysregulated levels might exacerbate autoimmunity and be detrimental to inflammatory bowel diseases.^52^ In this respect, we provide the molecular basis and foundation for the generation of the next class of probiotics.

## Materials and methods

### Bacterial strains and growth conditions

The lactic acid bacteria (LAB) species and strains used in this study are listed in Extended Data Table 1. *Lactobacillaceae* were routinely grown in a microaerophilic environment at 37 °C on solid or liquid Man-Ragosa-Sharpe (MRS) medium (Oxoid) without aeration. *Lactococcus lactis* (LL) strains were grown at 30 °C in M17 medium (Oxoid) supplemented with 0.5% glucose (GM17). When necessary, MRS and GM17 were supplemented with chloramphenicol at 8 and 5  µg ml^−1^, respectively. To construct fluorescently tagged strains, the plasmid pNZ-TuR-mCherry^13^ was introduced into competent cells of *Lactiplantibacillus plantarum* (LP) by electroporation as previously described.^53^ All bacterial isolates were maintained at −80 °C frozen stocks in their appropriate media with a 12.5% final concentration of glycerol.

### Self-aggregation and hydrophobicity assays

The self-aggregation and hydrophobicity of the LP cells were quantified according to Zhang et al. ^54^ but with some modifications. Briefly, overnight cultures were harvested by centrifugation at 5000 × g for 10 min, washed twice with PBS and resuspended in PBS to an OD_600_ of 0.5–0.6. The self-aggregation assay was carried out by aliquoting 4 mL of the suspension into tubes that were vortexed and left undisturbed at 37 °C for 5 h. The OD_600_ of the upper fraction (~1 mL) was then recorded to calculate the self-aggregation percentage using the following formula: 1-(A_5_/A_0_) × 100, where A_0_ and A_5_ represent the OD_600_ readings at 0 and 5 h, respectively. Similarly, the hydrophobicity assay was carried out by adding a 3 mL suspension to 1 mL of xylene (Fisher chemicals) and vortexing until homogenization. After a 30 min incubation at 37 °C, the OD_600_ of the aqueous phase was recorded to calculate the cell surface hydrophobicity percentage using the following formula: 1−(A/A_0_) × 100, where A_0_ and A are the OD_600_ readings at 0 and 30  min, respectively.

### Cell cultures: propagation, differentiation and transfection

Human THP-1 reporter monocyte-like cells and their corresponding macrophage-like cells were used as a model to investigate LP phagocytosis. As both type of cells behave as phagocytes, they were referred to as monocytes and macrophages in the manuscript. The monocytic cells included: (1) THP-1-IFIT1-Gaussia luciferase (Gluc); ^13^ (2) THP-1-IFIT1-Gluc deficient for STING or MAVS (KO STING or MAVS THP-1-IFIT1-Gluc); ^55^ (3) THP-1 Dual™-NF-kB-Blue/IFIT-2-Lucia luciferase (Lluc) (InvivoGen); and (4) THP-1 Dual™-NF-κB-Blue/IFIT-2-Lluc deficient for IFNAR2 or cGAS (KO MyD88 or cGAS THP-1-Dual™) (InvivoGen). In these cell lines, Gluc or Lluc are expressed and secreted under the control of the promoter of the interferon-induced protein with tetratricopeptide repeats 1 (IFIT1) or 2 (IFIT2), and blue secreted embryonic alkaline phosphatase (SEAP) is driven by NF-κB activation. In all cases, the cells were propagated in complete media of Roswell Park Memorial Institute (RPMI) 1640 (Life Technologies) supplemented with 15% fetal bovine serum (FBS, Seralab) and 1% penicillin/streptomycin (Pen/Strep, Life Technologies) at 37 °C in an atmosphere of 5% CO_2_. Differentiation of THP-1 monocytes into macrophages was carried out using 20  ng/mL of phorbol 12-myristate 13-acetate (PMA, Santa Cruz Biotechnology) under normal incubation conditions for 48  h as previously described.^6^ When required, the resulting THP-1 macrophages were subjected to transient transfection using Opti-MEM medium (Life Technologies), Lipofectamine RNAi Max (Thermo Fisher) and MISSION® pre-designed short interfering RNA (siRNA) targeting NOD2 (Id, SASI_Hs02_00355593) (Merck) following the manufacturer’s instructions. Transfection efficiency was evaluated by immunofluorescence microscopy using the MISSION® siRNA Fluorescent Universal Negative Control #1, 6-FAM (6-FAM-siRNA) (Merck) followed by qPCR to validate the knockdown efficiency at 24 and 40 h post-transfection, respectively. Max Planck Institute (MPI) self-renewing primary macrophages derived from murine foetal liver were also used in this study and were propagated as described above but supplemented with 2% spent medium from murine GM-CSF transfected X-63 cells.^56^

### Macrophage challenge, reporter-based assays and cytokine detection

Macrophages were exposed to the LAB strains listed in Extended Data Table 1, and the IFIT-1/2 or NF-κB activation was recorded following protocols described previously.^6^ Briefly, PMA-differentiated THP-1 macrophages seeded in a 96-well plate at a density of 5 × 10^4^ were challenged with the LAB strains at a ratio of 50 cells per 1 macrophage in RPMI (with 2% FBS) without Pen/Strep and incubated at 37 °C, 5% CO_2_ for 2 h before being replaced with fresh RPMI with 1% Pen/Strep and re-incubated for an additional 22 h. To ensure consistency in the macrophage stimulation, all bacterial challenges were carried out with equivalent levels of viable cell counts and total protein concentrations (Extended Data Figure 1). Where necessary, bacterial cells were simultaneously exposed to antibiotics (ampicillin at 0.5  μg/mL, chloramphenicol at 4  μg/mL or erythromycin at 1  μg/mL) or previously treated in PBS with heat (at 70 °C for 2 h) or proteases (trypsin at 25  μg/mL or proteinase K at 100  μg/mL for 1 h at 37 °C). Exposure to antibiotics was conducted with MICs, while the concentrations for the proteases were optimized to allow bacterial survival. The JAK1 inhibitors Ruxolitinib (RUX) and 6P were tested at the concentrations given in their corresponding figures, while the TRIF inhibitor was dissolved in DMSO and used at 40 μM. The positive controls (IFN-I inducers) were cells either treated with IFN-*β* at 5 ng/mL, cGAMP at 30 µg/mL, or LPS at 0.2 mg/mL or transfected with HT-DNA at 0.5  μg/mL using Lipofectamine 2000 (Thermo Fisher) and Opti-MEM according to the manufacturer’s instructions. The supernatants were then collected from the stimulated macrophages and transferred to a white-bottom 96-well plate for luciferase (Gluc/Lluc) measurement on a FLUOstar® Omega plate reader (BMG Labtech) following the addition of native coelenterazine (Thermo Scientific) at 2 µg/mL. Based on the luminescence response, the activation of IFIT1/IFT2 was calculated as the fold increase ± SD over the measurements for unchallenged macrophages. The collected supernatants were also used to detect and quantify human IFN-*β* and TNF-*α* production at 8 h post-LP exposure using the Invitrogen Human TNF-*α* Uncoated ELISA kit, and the Elabscience Human IFN-*β* ELISA kit, respectively. A time-course analysis conducted in our previous work ^6^ showed that the highest production of IFN-*β* in LP-challenged macrophages occurred after 8 h of incubation. This timepoint is also sufficient to observe considerable production of TNF-*α.*^57^ The absorbance was read on a CLARIOstar microplate reader (BMG Labtech) according to the ELISA kits’ manufactures’ instructions.

### Flow cytometry analysis

THP-1 or MPI macrophages were seeded in a 6-well plate at 10^6^ cells per well and exposed to mCherry-expressing LPs or LL labeled with fluorescein isothiocyanate (FITC, Sigma) at 100  mg/mL for 15  min in darkness at a ratio of 50:1 (bacteria:macrophage) for 2 h at 37 °C and 5% CO_2_. The macrophages were then washed twice with PBS to remove unbound bacteria, detached with 1.5  mM ethylenediaminetetraacetic acid (EDTA) in PBS (without calcium and magnesium) and resuspended in 2 volumes of the same solution to achieve a final cell concentration of 0.5 × 10^6^ cells/mL. The samples were run on the Attune™ NxT flow cytometer (Thermo Fisher), and the resulting data were analyzed with the FlowJo software to detect and quantify the levels of mCherry or FITC in the challenged macrophages in the appropriate fluorescence channel, thereby determining bacterial interaction with the macrophages and vice versa (Extended Data Figure 3).

### Immunofluorescence staining and imaging

THP-1 or MPI macrophages were seeded in a 96 clear bottom black well plate and simultaneously stained with Hoechst 33342 (Invitrogen) at 1 µg/mL and CellMask Green Tracking stains (Invitrogen) at a 1x concentration in complete media for 30 min for the detection of nuclei and F-actin, respectively. The cells were then challenged with the mCherry-expressing LP strains at a ratio of 10 bacteria per 1 macrophage for 2 h at 37 °C, 5% CO_2_, washed with PBS (without calcium and magnesium) and fixed for 30  min with 4% paraformaldehyde (PFA). After a final washing step with PBS, images were acquired using the Cytation V cell imaging multi-mode reader (Agilent Technologies) and processed with ImageJ.

### RNA extraction and transcriptional analysis

RNA was extracted from macrophages previously exposed to LP using the High Pure RNA Isolation Kit (Roche) and reverse transcribed using the SuperScript® III First-Strand Synthesis System (Invitrogen). The resulting cDNA was diluted 1:5 in water to be used as a template for real-time amplification on a QuantStudio 7 Flex Real-Time PCR system (Applied Biosystems) using SYBR Green Master Mix (Applied Biosystems) and specific primers (Extended Data Table 2). The expression of the target genes was first normalized using a house-keeping gene and then compared to that from non-stimulated control cells to yield a fold induction. Total RNA was also extracted from the LP strains in their mid-log growth phase using the same kit as indicated above but with an initial 30-min incubation step with lysozyme at 30 mg/mL to disrupt the cell wall. The extracted RNA was subjected to quality control based on its purity, concentration and integrity number using a bioanalyzer (Agilent) and sent to Novogene for whole-transcriptome sequencing. Briefly, cDNA libraries were constructed from samples containing RNA at a concentration of 1.0 ng/mL and paired-end sequenced using an Illumina TruSeq™ on an Illumina HiSeq 4000 platform. Upon mapping to the reference genome of the LP strain WCFS-1, sequences from each of the generated libraries were annotated and subjected to differential gene expression analysis using DESeq2. The resulting differentially expressed genes (DEGs) were then grouped into different biological processes, terms or pathways using the enrichment analysis database Gene Ontology (GO), Kyoto Encyclopedia of Genes and Genomes (KEGG) and the protein‒protein interaction (PPI) network.

### Extraction of cell wall proteins

Cell wall proteins were extracted from LP cells according to methods previously reported,^58^ but with some modifications. The cells from the LP cultures were first harvested by centrifuging at 7500 × g for 10  min, washed twice with ice-cold distilled water, and resuspended in 5 M LiCl at 20% of the initial culture volume. Following a 1-h incubation at 175 rpm and 37 °C, the resulting LiCl-treated cells were centrifuged at 10,000 × g for 20  min at 10 °C, washed twice with PBS, and resuspended in PBS at the initial culture volume. The cells were then subjected to further analysis, including analysis of macrophage uptake by flow cytometry and IFIT1 activation in THP1 macrophages. Additionally, the supernatants following the LiCl treatment were pooled together with samples from the first PBS wash and filter sterilized (0.22 μm pore size). LP cells were also lysed using B-PER™ Reagent (Thermo Fisher) according to the manufacturer’s instructions, but including a pre-treatment step with lysozyme at 30 mg/mL. Both the pooled supernatant samples and lysates were concentrated by acetone precipitation according to protocols adapted from Thermo Fisher Tech (Tip #4951), vigorously vortexed with two volumes of cold acetone (−20 °C) and left at −20 °C overnight. Pellets were finally obtained after centrifugation at 10,000 × g for 10 min at 4 °C, air dried for 10 min, resuspended in MilliQ water at a final concentration of 3 mg/mL and stored for further use as indicated below.

### SDS–PAGE and proteomics

Concentrated samples from the pooled supernatants and lysates were diluted in 4x Laemmli sample buffer (Bio-Rad) with *β*-mercaptoethanol at a 3:1 ratio, denatured at 95 °C for 5  min and resolved by SDS-PAGE on an 8%–16% Mini-PROTEAN TGX gel (Bio-Rad). The gels were stained with ReadyBlue™ Protein Gel Coomassie stain (Sigma) as specified by the manufacturer’s protocol, washed with reverse osmosis (RO) water and then visualized on a GelDoc EZ System (Bio-Rad). Bands of interest were then excised from the gel and, together with the concentrated pooled supernatants, subjected to an overnight trypsinization at 37 °C for peptide separation and further identification and quantification by nano-LC–MS as previously described.^59^ Liquid chromatography and mass spectrometry were performed using a nano RSLC Dionex U3000 system fitted to a Q-Exactive mass spectrometer (Thermo Scientific, San Jose, United States). The X!TademPipeline software was used to identify peptides from the MS/MS spectra, which were searched against the proteins encoded by the genomes of the LP strains P5 (LP^−^), WCFS-1 (LP^+^) and EML1 (LP^++^) (GenBank accessions no. GCA_011009755.1, GCA_000203855.3 and GCA_008016845.1, respectively). The database search parameters included: (i) the trypsin enzyme cleavage; (ii) a peptide mass tolerance of 10 ppm for MS and 0.05 Da for MS/MS; and (iii) oxidation of methionine and phosphorylation on threonine, serine and tryptophan as variable modifications. For each peptide identified, a minimum score corresponding to an e-value below 0.05 was selected as a prerequisite within a minimum number of 2 peptides per protein. A protein was considered present when it was detected in at least two of the three biological replicates. Each peptide that was identified by tandem MS was quantified using the i2MassChroq v.1.0.7 software.

### Immunoblotting

LP-challenged THP-1 macrophages were treated with RIPA buffer containing 1X protease and 1X phosphatase inhibitors (Roche) and benzonase (Sigma) at 250 U/ml for 30 min at 4 °C as previously described.^6^ Lysates were then denatured for 5 min at 95 °C in the presence of loading buffer, resolved by SDS–PAGE and transferred to nitrocellulose membranes (GE Healthcare) using a Trans-Blot semidry transfer unit (Bio-Rad). The resulting membranes were blocked in 0.05% Triton X-100/phosphate-buffered saline supplemented with 0.1% skimmed milk (Sigma) and subjected to immunoblotting with the following primary antibodies at the indicated dilutions: phosphorylated TBK-1 Ser172 (Cell signaling; 1:1000), TBK-1 (Cell signaling; 1:1000), phosphorylated IRF3 (Cell signaling; 1:1000), IRF3 (Cell signaling; 1:1000), IRF7 (Cell signaling; 1:1000), phosphorylated STAT1 (Cell signaling; 1:1000), STAT1 (Cell signaling; 1:1000), STING (Cell signaling; 1:1000), cGAS (Cell signaling; 1:1000) and *α*-tubulin (Cell signaling, 1:1000). Primary antibodies were detected using IRDye-conjugated secondary antibodies via an Odyssey infrared imager (LI-COR Biosciences). Images were analyzed using the software Image Studio, including quantitative analysis of the total protein expression ratio between the target protein and *α*-tubulin (housekeeping protein).

### Statistical analysis

Statistical analysis was performed using GraphPad Prism, and unless stated otherwise, the data are presented as the means ± standard deviation (SD) of at least three independent biological replicates. Significant differences between one sample and its corresponding control were determined using the Student’s t-test and, within a group of samples, one-way ANOVA was used, followed by Fisher’s least significant difference (LSD) test.

## Supplementary Material

Extended Data Excel File 1.xlsx Extended Data Excel File 1.xlsx

Extended Data Excel File 2.xlsx Extended Data Excel File 2.xlsx

Supplementary material Extended_Data

## Data Availability

The RNA-seq data are available in the SRA under BioProject accession number PRJNA1208499. The mass spectrometry proteomics data have been deposited in the ProteomeXchange Consortium via the PRIDE partner repository with the dataset identifier PXD063717. Source data are provided with this paper.

## Additional information

**Extended data** is available for this paper.

**Correspondence and requests for materials** should be addressed to Jorge Gutierrez-Merino.

## References

[1] Belkaid Y, Hand TW. Role of the microbiota in immunity and inflammation. Cell. 2014;157:121–141. doi: 10.1016/j.cell.2014.03.011. 24679531 PMC4056765

[2] Martens EC, Neumann M, Desai MS. Interactions of commensal and pathogenic microorganisms with the intestinal mucosal barrier. Nat Rev Microbiol. 2018;16:457–470. doi: 10.1038/s41579-018-0036-x. 29904082

[3] McAleer JP, Kolls JK. Contributions of the intestinal microbiome in lung immunity. Eur J Immunol. 2018;48:39–49. doi: 10.1002/eji.201646721. 28776643 PMC5762407

[4] Wells JM. Immunomodulatory mechanisms of lactobacilli. Microb Cell Fact. 2011;10(Suppl 1):S17. doi: 10.1186/1475-2859-10-S1-S17. 21995674 PMC3231924

[5] Kim CH. Immune regulation by microbiome metabolites. Immunology. 2018;154:220–229. doi: 10.1111/imm.12930. 29569377 PMC5980225

[6] Gutierrez-Merino J, Isla B, Combes T, Martinez-Estrada F, De Motes CM. Beneficial bacteria activate type-I interferon production via the intracellular cytosolic sensors STING and MAVS. Gut Microbes. 2020;11:771–788. doi: 10.1080/19490976.2019.1707015. 31941397 PMC7524384

[7] Motwani M, Pesiridis S, Fitzgerald KA. DNA sensing by the cGAS-STING pathway in health and disease. Nat Rev Genet. 2019;20:657–674. doi: 10.1038/s41576-019-0151-1. 31358977

[8] Trinchieri G. Type I interferon: friend or foe?. J Exp Med. 2010;207:2053–2063. doi: 10.1084/jem.20101664. 20837696 PMC2947062

[9] Schoggins JW, Rice CM. Interferon-stimulated genes and their antiviral effector functions. Curr Opin Virol. 2011;1:519–525. doi: 10.1016/j.coviro.2011.10.008. 22328912 PMC3274382

[10] Panigrahi P, Parida S, Nanda NC, Satpathy R, Pradhan L, Chandel DS, Baccaglini L, Mohapatra A, Misra PR, Chaudhry R, et al. A randomized synbiotic trial to prevent sepsis among infants in rural India. Nature. 2017;548:407–412. doi: 10.1038/nature23480. 28813414

[11] Gutierrez-Castrellon P, Gandara-Marti T, Abreu YAAT, Nieto-Rufino CD, Lopez-Orduna E, Jimenez-Escobar I, Gutiérrez-Castrellón P, Gandara-Martí T, Abreu Y Abreu AT, López-Orduña E, et al. Probiotic improves symptomatic and viral clearance in Covid19 outpatients: a randomized, quadruple-blinded, placebo-controlled trial. Gut Microbes. 2022;14:2018899. doi: 10.1080/19490976.2021.2018899. 35014600 PMC8757475

[12] Diamond MS, Farzan M. The broad-spectrum antiviral functions of IFIT and IFITM proteins. Nat Rev Immunol. 2013;13:46–57. doi: 10.1038/nri3344. 23237964 PMC3773942

[13] Mankan AK, Schmidt T, Chauhan D, Goldeck M, Honing K, Gaidt M, Höning K, Kubarenko AV, Andreeva L, Hopfner K, et al. Cytosolic RNA:DNA hybrids activate the cGAS-STING axis. EMBO J. 2014;33:2937–2946. doi: 10.15252/embj.201488726. 25425575 PMC4282641

[14] Stedman A, van Vliet AHM, Chambers MA, Gutierrez-Merino J. Gut commensal bacteria show beneficial properties as wildlife probiotics. Ann NY Acad Sci. 2020;1467:112–132. doi: 10.1111/nyas.14302. 32026493

[15] Kleerebezem M, Boekhorst J, van Kranenburg R, Molenaar D, Kuipers OP, Leer R, Tarchini R, Peters SA, Sandbrink HM, Fiers MWEJ, et al. Complete genome sequence of *Lactobacillus plantarum* WCFS1. Proc Natl Acad Sci U S A. 2003;100:1990–1995. doi: 10.1073/pnas.0337704100. 12566566 PMC149946

[16] Bravo M, Combes T, Martinez FO, Risco D, Goncalves P, Garcia-Jimenez WL, Gonçalves P, Cerrato R, Fernandez-Llario P, Gutierrez-Merino J, et al. Wildlife symbiotic bacteria are indicators of the health status of the host and its ecosystem. Appl Environ Microbiol. 2022;88:e0138521. doi: 10.1128/AEM.01385-21. 34669453 PMC8752132

[17] Mathiesen G, Overland L, Kuczkowska K, Eijsink VGH. Anchoring of heterologous proteins in multiple *lactobacillus* species using anchors derived from *lactobacillus* plantarum. Sci Rep. 2020;10:9640. doi: 10.1038/s41598-020-66531-7. 32541679 PMC7295990

[18] Michon C, Langella P, Eijsink VGH, Mathiesen G, Chatel JM. Display of recombinant proteins at the surface of lactic acid bacteria: strategies and applications. Microb Cell Fact. 2016;15:70. doi: 10.1186/s12934-016-0468-9. 27142045 PMC4855500

[19] Holst B, Glenting J, Holmstrom K, Israelsen H, Vrang A, Antonsson M, Holmstrøm K, Ahrné S, Madsen SM, Dudley EG. Molecular switch controlling expression of the mannose-specific adhesin, MSA, in *lactobacillus plantarum*. Appl Environ Microbiol. 2019;85:e02954-18. doi: 10.1128/AEM.02954-18. 30877113 PMC6498163

[20] Gasson MJ. Plasmid complements of *Streptococcus lactis* Ncdo-712 and other lactic streptococci after protoplast-induced curing. J Bacteriol. 1983;154:1–9. doi: 10.1128/jb.154.1.1-9.1983. 6403500 PMC217423

[21] Stedman A, Chambers MA, Gutierrez-Merino J. Secretion and functional expression of *mycobacterium bovis* antigens MPB70 and MPB83 in lactic acid bacteria. Tuberculosis. 2019;117:24–30. doi: 10.1016/j.tube.2019.05.007. 31378264

[22] Langa S, Peirotén A, Arqués JL, Landete JM. Evoglow-Pp1 and mCherry proteins: a dual fluorescent labeling system for lactic acid bacteria. Appl Microbiol Biot. 2021;105:7367–7378. doi: 10.1007/s00253-021-11537-y. 34536099

[23] Saito K, Tomita S, Nakamura T. Aggregation of *Lactobacillus brevis* associated with decrease in pH by glucose fermentation. Biosci Biotechnol Biochem. 2019;83:1523–1529. doi: 10.1080/09168451.2019.1584522. 30822234

[24] do Carmo FLR, Rabah H, Carvalho RDO, Gaucher F, Cordeiro BF, da Silva SH, De Oliveira Carvalho RD, Le Loir Y, Azevedo V, Jan G. Extractable bacterial surface proteins in probiotic-host interaction. Front Microbiol. 2018;9:645. doi: 10.3389/fmicb.2018.00645. 29670603 PMC5893755

[25] Sengupta R, Altermann E, Anderson RC, McNabb WC, Moughan PJ, Roy NC. The role of cell surface architecture of lactobacilli in host-microbe interactions in the gastrointestinal tract. Mediators Inflamm. 2013;2013:237921. doi: 10.1155/2013/237921. 23576850 PMC3610365

[26] Biswas A, Petnicki-Ocwieja T, Kobayashi KS. Nod2: a key regulator linking microbiota to intestinal mucosal immunity. J Mol Med (Berl). 2012;90:15–24. doi: 10.1007/s00109-011-0802-y. 21861185 PMC3263373

[27] Sun LJ, Wu JX, Du FH, Chen X, Chen ZJJ. Cyclic GMP-AMP synthase is a cytosolic DNA sensor that activates the type I interferon pathway. Sci. 2013;339:786–791. doi: 10.1126/science.1232458. PMC3863629 23258413

[28] Burdette DL, Monroe KM, Sotelo-Troha K, Iwig JS, Eckert B, Hyodo M, Hayakawa Y, Vance RE. STING is a direct innate immune sensor of cyclic di-GMP. Nature. 2011;478:515–518. doi: 10.1038/nature10429. 21947006 PMC3203314

[29] McNab F, Mayer-Barber K, Sher A, Wack A, O'Garra A. Type I interferons in infectious disease. Nat Rev Immunol. 2015;15:87–103. doi: 10.1038/nri3787. 25614319 PMC7162685

[30] Ivashkiv LB, Donlin LT. Regulation of type I interferon responses. Nat Rev Immunol. 2014;14:36–49. doi: 10.1038/nri3581. 24362405 PMC4084561

[31] Wirusanti NI, Baldridge MT, Harris VC. Microbiota regulation of viral infections through interferon signaling. Trends Microbiol. 2022;30:778–792. doi: 10.1016/j.tim.2022.01.007. 35135717 PMC9344482

[32] Kawashima T, Kosaka A, Yan H, Guo Z, Uchiyama R, Fukui R, Kaneko D, Kumagai Y, You D, Carreras J, et al. Double-stranded RNA of intestinal commensal but not pathogenic bacteria triggers production of protective interferon-b. eta. Immunity. 2013;38:1187–1197. doi: 10.1016/j.immuni.2013.02.024. 23791646

[33] Weiss G, Maaetoft-Udsen K, Stifter SA, Hertzog P, Goriely S, Thomsen AR, Paludan SR, Frøkiær H. MyD88 drives the IFN-β response to *Lactobacillus acidophilus* in dendritic cells through a mechanism involving IRF1, IRF3, and IRF7. J Immunol. 2012;189:2860–2868. doi: 10.4049/jimmunol.1103491. 22896628

[34] Si W, Liang H, Bugno J, Xu Q, Ding X, Yang K, Fu Y, Weichselbaum RR, Zhao X, Wang L. *Lactobacillus rhamnosus* GG induces cGAS/STING- dependent type I interferon and improves response to immune checkpoint blockade. Gut. 2022;71:521–533. doi: 10.1136/gutjnl-2020-323426. 33685966 PMC8710942

[35] Duar RM, Lin XB, Zheng J, Martino ME, Grenier T, Perez-Munoz ME, Pérez-Muñoz ME, Leulier F, Gänzle M, Walter J. Lifestyles in transition: evolution and natural history of the genus *lactobacillus*. FEMS Microbiol Rev. 2017;41:S27–S48. doi: 10.1093/femsre/fux030. 28673043

[36] Garcia-Gonzalez N, Prete R, Battista N, Corsetti A. Adhesion properties of food-associated *Lactobacillus plantarum* strains on human intestinal epithelial cells and modulation of IL-8 release. Front Microbiol. 2018;9:2392. doi: 10.3389/fmicb.2018.02392. 30349520 PMC6186789

[37] Isenring J, Geirnaert A, Lacroix C, Stevens MJA. Bistable auto-aggregation phenotype in *Lactiplantibacillus plantarum* emerges after cultivation in in vitro colonic microbiota. BMC Microbiol. 2021;21:268. doi: 10.1186/s12866-021-02331-x. 34610822 PMC8493755

[38] Gorreja F, Walker WA. The potential role of adherence factors in probiotic function in the gastrointestinal tract of adults and pediatrics: a narrative review of experimental and human studies. Gut Microbes. 2022;14:2149214. doi: 10.1080/19490976.2022.2149214. 36469568 PMC9728474

[39] Gomez NC, Ramiro JM, Quecan BX, de Melo Franco BD. Use of potential probiotic lactic acid bacteria (LAB) biofilms for the control of *Listeria monocytogenes*, *Salmonella typhimurium*, and *Escherichia coli* O157:H7 biofilms formation. Front Microbiol. 2016;7:863. doi: 10.3389/fmicb.2016.00863. 27375584 PMC4901071

[40] Wang Y, Goossens E, Eeckhaut V, Perez Calvo E, Lopez-Ulibarri R, Eising I, Pérez Calvo E, Klausen M, Debunne N, De Spiegeleer B, et al. Dietary muramidase degrades bacterial peptidoglycan to NOD-activating muramyl dipeptides and reduces duodenal inflammation in broiler chickens. Br J Nutr. 2021;126:641–651. doi: 10.1017/S0007114520004493. 33172510

[41] Shu C, Li X, Li P. The mechanism of double-stranded DNA sensing through the cGAS-STING pathway. Cytokine Growth Factor Rev. 2014;25:641–648. doi: 10.1016/j.cytogfr.2014.06.006. 25007740 PMC4254336

[42] Hwang HS, Lee MH, Choi MH, Kim HA. Induction of pro-inflammatory cytokines by 29-kDa FN-f via cGAS/STING pathway. BMB Rep. 2019;52:336–341. doi: 10.5483/BMBRep.2019.52.5.072. 31068249 PMC6549918

[43] Pandey AK, Yang YB, Jiang ZZ, Fortune SM, Coulombe F, Behr MA, Fitzgerald KA, Sassetti CM, Kelliher MA, Cossart P. NOD2, RIP2 and IRF5 play a critical role in the type I interferon response to. PLoS Pathog. 2009;5:e1000500. doi: 10.1371/journal.ppat.1000500. 19578435 PMC2698121

[44] Xie J, Cheng J, Ko H, Tang Y. Cytosolic DNA sensors in neurodegenerative diseases: from physiological defenders to pathological culprits. EMBO Mol Med. 2024;16:678–699. doi: 10.1038/s44321-024-00046-w. 38467840 PMC11018843

[45] Charrel-Dennis M, Latz E, Halmen KA, Trieu-Cuot P, Fitzgerald KA, Kasper DL, Golenbock DT. TLR-independent type I interferon induction in response to an extracellular bacterial pathogen via intracellular recognition of its DNA. Cell Host Microbe. 2008;4:543–554. doi: 10.1016/j.chom.2008.11.002. 19064255 PMC3727391

[46] Lancaster CE, Fountain A, Dayam RM, Somerville E, Sheth J, Jacobelli V, Terebiznik MR, Botelho RJ. Phagosome resolution regenerates lysosomes and maintains the degradative capacity in phagocytes. J Cell Biol. 2021;220(9): e202005072. doi: 10.1083/jcb.202005072. 34180943 PMC8241537

[47] Mogensen TH. IRF and STAT transcription factors - from basic biology to roles in infection, protective immunity, and primary immunodeficiencies. Front Immunol. 2018;9:3047. doi: 10.3389/fimmu.2018.03047. 30671054 PMC6331453

[48] Ma F, Li B, Yu Y, Iyer SS, Sun M, Cheng G. Positive feedback regulation of type I interferon by the interferon-stimulated gene STING. EMBO Rep. 2015;16:202–212. doi: 10.15252/embr.201439366. 25572843 PMC4328747

[49] Metzemaekers M, Vanheule V, Janssens R, Struyf S, Proost P. Overview of the mechanisms that May contribute to the non-redundant activities of interferon-inducible CXC chemokine receptor 3lLigands. Front Immunol. 2017;8:1970. doi: 10.3389/fimmu.2017.01970. 29379506 PMC5775283

[50] Lozhkov AA, Klotchenko SA, Ramsay ES, Moshkoff HD, Moshkoff DA, Vasin AV, Salvato MS. The key roles of interferon lambda in human molecular defense against respiratory viral infections. Pathogens. 2020;9:989. doi: 10.3390/pathogens9120989. 33255985 PMC7760417

[51] Di Domizio J, Blum A, Gallagher-Gambarelli M, Molens JP, Chaperot L, Plumas J. TLR7 stimulation in human plasmacytoid dendritic cells leads to the induction of early IFN-inducible genes in the absence of type I IFN. Blood. 2009;114:1794–1802. doi: 10.1182/blood-2009-04-216770. 19553637 PMC2847942

[52] Yang Y, Wang L, Peugnet-Gonzalez I, Parada-Venegas D, Dijkstra G, Faber KN. cGAS-STING signaling pathway in intestinal homeostasis and diseases. Front Immunol. 2023;14:1239142. doi: 10.3389/fimmu.2023.1239142. 37781354 PMC10538549

[53] Aukrust T, Blom H. Transformation of *lactobacillus* strains used in meat and vegetable fermentations. Food Res Int. 1992;25:253–261. doi: 10.1016/0963-9969(92)90121-K.

[54] Zhang B, Zuo FL, Yu R, Zeng Z, Ma HQ, Chen SW. Comparative genome-based identification of a cell wall-anchored protein from *lactobacillus plantarum* increases adhesion of *lactococcus lactis* to human epithelial cells. Sci Rep. 2015;5:14109. doi: 10.1038/srep14109. 26370773 PMC4572922

[55] Tie CH, Fernandes L, Conde L, Robbez-Masson L, Sumner RP, Peacock T, Robbez‐Masson L, Rodriguez‐Plata MT, Mickute G, Gifford R, et al. KAP1 regulates endogenous retroviruses in adult human cells and contributes to innate immune control. EMBO Rep. 2018;19, 10.15252/embr.201745000. PMC6172469 30061100

[56] Fejer G, Wegner MD, Györy I, Cohen I, Engelhard P, Voronov E, Manke T, Ruzsics Z, Dölken L, Prazeres da Costa O, et al. Nontransformed, GM-CSF-dependent macrophage lines are a unique model to study tissue macrophage functions. Proc Natl Acad Sci U S A. 2013;110:E2191–E8. doi: 10.1073/pnas.1302877110. 23708119 PMC3683787

[57] Stedman A, Maluquer de Motes C, Lesellier S, Dalley D, Chambers M, Gutierrez-Merino J. Lactic acid bacteria isolated from european badgers (*meles meles*) reduce the viability and survival of *bacillus* calmette-guerin (BCG) vaccine and influence the immune response to BCG in a human macrophage model. BMC Microbiol. 2018;18:74. doi: 10.1186/s12866-018-1210-z. 30005620 PMC6044090

[58] Zhang YC, Zhang LW, Tuo YF, Guo CF, Yi HX, Li JY, Han X, Du M. Inhibition of *shigella sonnei* adherence to HT-29 cells by lactobacilli from Chinese fermented food and preliminary characterization of S-layer protein involvement. Res Microbiol. 2010;161:667–672. doi: 10.1016/j.resmic.2010.06.005. 20600857

[59] Tarnaud F, Gaucher F, do Carmo FLR, Illikoud N, Jardin J, Briard-Bion V, Guyomarc’h F, Gagnaire V, Jan G. Differential adaptation of *propionibacterium freudenreichii* CIRM-BIA129 to cow's milk versus soymilk environments modulates its stress tolerance and proteome. Front Microbiol. 2020;11:549027. doi: 10.3389/fmicb.2020.549027. 33335514 PMC7736159

