# Supplementary material for: Self-aggregating Lactiplantibacillus plantarum enhances type-I interferon responses via the cytosolic sensors NOD2 and cGAS
Source: Gut Microbes. 2026 Jan 28;18(1):2615490. doi: 10.1080/19490976.2026.2615490 (PMC12854382; doi:10.1080/19490976.2026.2615490)
Supplement: Supplementary material — Extended_Data [file KGMI_A_2615490_SM1430.docx]

Extended Data Table 1. Bacteria and plasmids used in this study.

| Strain or plasmid | Internal nomenclature and relevant characteristics | Source or reference |
| --- | --- | --- |
| Strains |  |  |
| *Lactobacillus acidophilus* 637 | *L. acidophilus* | Yoghurt |
| *Levilactobacillus brevis* NCIB 8038 | *L. brevis* | Fermented olives |
| *Latilactobacillus curvatus* A/B 860 | *L. curvatus* | Cured meat product |
| *Apilactobacillus kunkeei* N16 | *L. kunkeei* | Honeybee pollen |
| *Lacticaseibacillus paracasei* DN-114001 | *L. paracasei* 1 | Probiotic drink |
| *Lacticaseibacillus paracasei* Shirota | *L. paracasei* 2 | Probiotic drink |
| *Lacticaseibacillus paracasei* SA5 | *L. paracasei* 3 | Wild boar |
| *Lactiplantibacillus plantarum* P5 | *L. plantarum* 1 (LP^-^) | European badger |
| *Lactiplantibacillus plantarum* P5-mCherry | LP^-^ -mCherry, Recombinant P5 strain expressing red fluoresence, Cm^R^ | This study |
| *Lactiplantibacillus plantarum* WCFS-1 | *L. plantarum* 2, (LP^+^) | Human saliva |
| *Lactiplantibacillus plantarum* WCFS-1.mCherry | LP^+^-mCherry, Recombinant WCFS-1 strain expressing red fluoresence, Cm^R^ | Langa S., et al (2021) |
| *Lactiplantibacillus plantarum* WCSF-1 AMJ1632 | W-32, Highly msa-expressing WCFS1 variant | Holst B., et al (2019) |
| *Lactiplantibacillus plantarum* WCFS-1 AMJ1633 | W-33, Highly msa-expressing WCFS1 variant | Holst B., et al (2019) |
| *Lactiplantibacillus plantarum* WCFS1 AMJ1634 | W-34, Highly msa-expressing WCFS1 variant | Holst B., et al (2019) |
| *Lactiplantibacillus plantarum* WCFS1 AMJ1635 | W-35, Highly msa-expressing WCFS1 variant | Holst B., et al (2019) |
| *Lactiplantibacillus plantarum*  NCIMB 30375 | *L. plantarum* 3 | Probiotic supplement |
| *Lactiplantibacillus plantarum* C1 | *L. plantarum* 4 | Wild boar |
| *Lactiplantibacillus plantarum* EML1 | *L. plantarum* 5, (LP^++^) | Wild boar |
| *Lactiplantibacillus plantarum* EML1-mCherry | LP^++^-mCherry, Recombinant EML1 strain expressing red fluoresence, Cm^R^ | This study |
| *Lactiplantibacillus plantarum* SA3 | *L. plantarum* 6 | Wild boar |
| *Ligilactobacillus salivarius*  C2 | *L. salivarius* 1 | Wild boar |
| *Ligilactobacillus salivarius*  C12 | *L. salivarius* 2 | Wild boar |
| *Limosilactobacillus reuteri*  DSM 20016 | *L. reuteri* 1 | Human intestine |
| *Limosilactobacillus reuteri*  DSM 17938 | *L. reuteri* 2 | Probiotic supplement |
| *Limosilactobacillus reuteri*  D4 | *L. reuteri* 3 | European badger |
| *Lactococcus lactis* MG1363 | *L. lactis* (LL), host for expression of *Lactobacillaceae* proteins (adhesin deficient) | Gasson MJ. (1983) |
| *Lactococcus lactis* MG1363-AMJ1296 | LL-msa, Recombinant MG1363 isolate expressing Mannose specific adhesin (msa) | Holst B., et al (2019) |
| Plasmids |  |  |
| pNZ:TuR.mCherry | pNZ8048 harbouring *mrfp* gene under the control of the elongation factor Tu promoter of *L. reuteri* DS20016 | Langa S., et al (2021) |

NCI(M)B: National Collections of Industrial (and Marine) Bacteria, Aberdeen, UK; DSM: Deutsche Sammlung von Mikroorganismen (German collection of microorganisms), Germany

Extended Data Table 2. Primers used in this study.

| **Gene** | **Target species** | **Sequence (5’🡪3’)** |
| --- | --- | --- |
| Glyceraldehyde-3-phosphate dehydrogenase (GAPDH, *gapB*) | *Lactiplantibacillus plantarum* | Fwd: CGGTGTTGACTTCGTTCTCG |
|  |  | Rv: CAGCAGTAACTTTCTTGTCTAA |
| Mannose specific adhesin (*msa*) | *Lactiplantibacillus plantarum* | Fwd: GCTCAGTTACCGGCACAAAA |
|  |  | Rv: TGTAGCTAGTGGTCAACGCA |
| GAPDH | Mice | Fwd: CATCACTGCCACCCAGAAGACTG |
|  |  | Rv: ATGCCAGTGAGCTTCCCGTTCAG |
| Interferon-beta (IFN-β) | Mice | Fwd: GCCTTTGCCATCCAAGAGATGC |
|  |  | Rv: ACACTGTCTGCTGGTGGAGTTC |
| GAPDH | Human | Fwd: ACCCAGAAGACTGTGGATGG |
|  |  | Rv: TTCTAGACGGCAGGTCAGGT |
| TATA box binding protein (TBP) | Human | Fwd: TGCACAGGAGCCAAGAGTGAA |
|  |  | Rv: CACATCACAGCTCCCCACCA |
| NOD2 | Human | Fwd: CGGCGTTCCTCAGGAAGTAC |
|  |  | Rv: ACCCCGGGCTCATGATG |
| Interferon beta (IFN-β) | Human | Fwd: ACATCCCTGAGGAGATTAAGCA |
|  |  | Rv: GCCAGGAGGTTCTCAACAATAG |
| Tumor necrosis factor alpha (TNF-α) | Human | Fwd: CCTGCTGCACTTTGGAGTGA |
|  |  | Rv: TCGAGAAGATGATCTGACTGCC |
| CXCL10 | Human | Fwd: TGGCATTCAAGGAGTACCTC |
|  |  | Rv: TTGTAGCAATGATCTCAACACG |
